# Altered peripapillary vessel density and nerve fiber layer thickness in thyroid-associated ophthalmopathy using optical coherence tomography angiography

**DOI:** 10.1007/s10792-021-02051-1

**Published:** 2021-10-12

**Authors:** Huan Jian, Yujiao Wang, Luyu Ou, Weimin He

**Affiliations:** Ophthalmic Laboratory, Department of Ophthalmology, West China Hospital, Sichuan University, No. 37 Guoxue Street, Chengdu, 610041 Sichuan China

**Keywords:** Thyroid-associated ophthalmopathy, Peripapillary vessel density, Retinal nerve fiber layer thickness, OCTA, Dysthyroid optic neuropathy

## Abstract

**Objective:**

To measure the peripapillary vessel density (VD) and retinal nerve fiber layer thickness (RNFLT) in thyroid-associated ophthalmopathy (TAO) and dysthyroid optic neuropathy (DON) patients using optical coherence tomography angiography (OCTA), and determine their prognostic relevance.

**Methods:**

Forty-three TAO patients with or without DON (82 eyes in total) and 26 healthy subjects (52 eyes) were enrolled. All participants underwent ophthalmology and endocrinology tests. The peripapillary VD in retinal peripapillary capillary layer and RNFLT were analyzed using OCTA images. Multiple linear regression analysis was used to assess the relationship between peripapillary VD /RNFLT and the relevant factors.

**Results:**

The total peripapillary VD and RNFLT were significantly lower in the DON patients compared to the other groups (*P* < 0.001, 95% confidence intervals), and each quadrant of VD and RNFLT showed similar results except temporal RNFLT. No significant difference was seen between the RNFLT and VD of active non-DON (ANDON), inactive non-DON (NDON) patients and normal control (NC) group. Multivariable linear regression model showed that high IOP is an independent risk factor for lower peripapillary VD and RNFLT (*β* = −0.465, *P* < 0.001 and *β* = −0.343, *P* = 0.002 respectively).

**Conclusion:**

OCTA parameters are suitable indicators for diagnosing DON. TAO patients with high IOP should be considered at high risk of retinal vessel and nerve fiber layer deterioration. In addition, patients with TAO should be advised to quit smoking since it could affect peripapillary VD and RNFLT.

## Introduction

Thyroid-associated ophthalmopathy (TAO) is an autoimmune disease that affects visual acuity, facial appearance and the quality of life. It is prevalent across all age groups, and the most severe cases are usually observed among older males [1]. The exact pathological basis of TAO is unclear at present, although immunological, genetic and environmental factors have been implicated in its onset and development [2]. Given its complex clinical features, a single diagnostic method cannot accurately evaluate disease severity. In addition, the current grading and staging systems of TAO, such as NOSPECS [3], CAS [4], VISA system [5] and EUGOGO system [6], are mostly subjective and difficult to follow in clinical practice. The onset of dysthyroid optic neuropathy (DON), a severe complication of TAO caused by optic nerve lesion subsequent to the increased orbital or intraocular pressure, is diagnosed on the basis of vision acuity damage, optic disk swelling or optic nerve compression by CT and MRI [7–9].

The current consensus is to improve the quantitative criteria for grading/staging TAO and diagnosing DON. Optical coherence tomography angiography (OCTA) is a non-invasive technique that can detect blood vessels of the retina and choroid through high-resolution and three-dimensional images [10], and measure vessel density (VD). OCTA images have shown that the retinal VD correlates negatively with the clinical activity score (CAS) but has no significant correlation with the NOSPECS classification [11, 12]. Studies show that compared to healthy individuals, TAO patients have lower retinal nerve fiber layer thickness (RNFLT) [13] and higher superficial VD in the macula area^10^. In addition, decreased peripapillary VD correlates with aggravated DON [10, 14]. However, another study did not find any significant difference between the macular nerve fiber layer thickness of TAO patients and normal controls [15]. The aim of our study was to determine the diagnostic utility of OCTA parameters for TAO and DON.

## Materials and methods

### Study design and subjects

Forty-three TAO patients (4 unilateral and 39 bilateral; 82 affected eyes in total, male-to-female ratio was 1: 1.48) were prospectively enrolled at the West China Hospital from November 2018 to March 2019 based on clinical, imaging and laboratory findings. TAO was diagnosed on the basis of Bartley’s criteria [16]: (1) patients with eyelid retraction presenting thyroid disorder, proptosis (more than 20 mm), optic nerve dysfunction of unknown cause, enlarged extraocular muscles that limit eye movement, or any combination of the above; (2) patients without lid retraction presenting thyroid dysfunction and proptosis, extraocular muscle involvement or optic nerve dysfunction. Based on the CAS at first examination, the TAO patients were further classified into active DON (ADON) or active non-DON (ANDON) group (CAS ≥ 3) and inactive NDON (NDON) group (CAS < 3) [6]. Subjects with other diseases that result in high intraocular pressure (IOP), ocular trauma or previous surgery, systemic metabolic or vascular disease (such as diabetes or hypertension), poor thyroid control, other systemic connective tissue disease or autoimmune disease, or previous TAO treatment with steroids or radiation were excluded. In addition, 26 healthy subjects (52 pairs of eyes, aged 19 to 59 years old, male to female ratio was 1: 1.6) were enrolled as the normal control (NC) group. The inclusion criterion was healthy individuals with normal vision acuity, while the individuals with any kind of ocular disease, high IOP, ocular trauma, previous eye surgery, thyroid disease, or systemic disease (such as metabolic, vascular, connective tissue or autoimmune disease) were excluded.

### Ocular assessment

Patients underwent regular ophthalmology tests, including best corrected visual acuity, IOP, exophthalmos, appearance, eye movement, slit lamp microscopy, fundus examination, perimetry, visual evoked potential examination, and enhancement scanning MRI of the ocular region. Best corrected visual acuity was tested using international standard logarithmic visual acuity chart. IOP was measured three times for each eye using TX-20 noncontact tonometer (Canon Corporation, Japan), and the average was calculated. Proptosis was measured using Hertel exophthalmometer.

### Optical coherence tomography angiography (OCTA) measurement

OCTA images were obtained using Optovue AngioVue™ (RTVue XR Avanti, Optovue Inc. Fremont, CA. USA) and analyzed with AngioAnalytics 2.0 quantization software, which uses the Split-Spectrum Amplitude Decorrelation Angiography algorithm. The wavelength was 840 nm, scanning frequency was 70,000 Hz, distinguishability of lateral and axial direction were 15 μm and 5 μm respectively, scanning depth was 2–3 mm, A-scan count was 304 × 304, and B-scan was repeated twice at the same spot. Motion Correction Technic and DualTrac were applied during the entire procedure. HD Angio Disc 4.5 mm mode was employed to scan a 4.5 × 4.5 mm area surrounding the optic nerve, and the retinal peripapillary capillary (ILM-NFL) layer was the default quantified vessel layer. Each position was measured twice, and peripapillary RNFLT and VD were recorded. All measurements were taken by one ophthalmologist.

### Statistical analysis

The data were analyzed using SPSS 23.0 software (SPSS Inc., Chicago, IL, USA IBM). Qualitative data were calculated as percentages and compared using Chi-square test or Fisher exact test. Quantitative data were calculated as mean ± standard deviation or medians and quartiles. One-way ANOVA with Bonferroni test was used to compare peripapillary RNFLT and nasal VD across multiple groups. Welch’s ANOVA with Games-Howell test was used for age, IOP, peripapillary VD and temporal RNFLT. Kruskal–Wallis test was used for duration and number of affected muscles. Factors significantly correlated with the peripapillary VD or RNFLT were identified by Spearman test or point-biserial correlation and incorporated into the multivariable linear regression model. P values < 0.05 were considered statistically significant.

## Results

### Baseline data and IOP

**Fig. 1:**
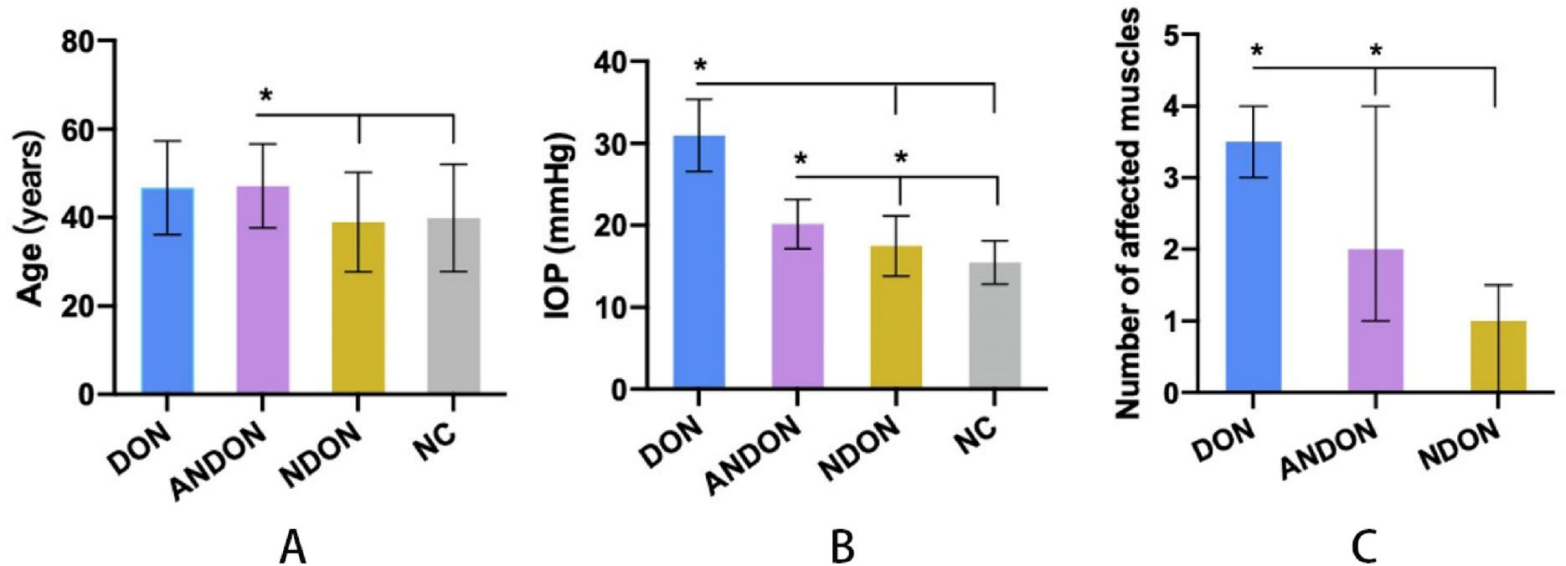
Evaluation of basic information including age **a**, IOP **b**, number of affected muscles **c** of the four groups. (*P < 0.05 between indicated groups)

**Fig. 2:**
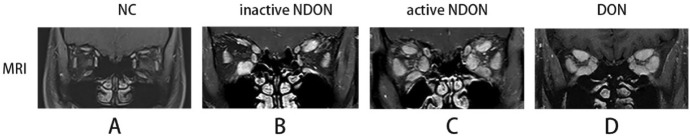
Coronal MRI pictures of the four groups. NC group showed no abnormal muscles. **a** NDON group. **b** and ANDON group. **c** showed varying degrees of enlarged extraocular muscles. DON group showed enlarged extraocular muscles and crowded orbital apex (**d**)

Eighty-two eyes of 43 TAO patients and 52 eyes of 26 control subjects were analyzed. The male-to-female ratio was 1:1.6 and 1:1.53, respectively in the NC and TAO groups, and the respective mean ages were 39.88 ± 12.17 (range 19–59) and 43.39 ± 11.09 (range 22 to 62) years. In the TAO group, 39 subjects (90.70%) were affected bilaterally, while 4 (9.30%) had unilateral condition. Furthermore, ADON was diagnosed for 6 eyes, whereas 37 eyes were NDON and 39 eyes were ANDON. Age and gender distribution was significantly different across the NC, ADON, ANDON and NDON groups (*p* = 0.007, 0.019, respectively). The median duration of ocular signs and TAO symptoms was 7.0 months (2–12 months, ranging from 1 to 120 months). In addition, 36 TAO patients (83.72%) had hyperthyroidism with median duration of 8.5 months (4.25–24.00 months, ranging from 1 to 120 months), and 10 (76.92%) of 13 patients undergoing I^131^ treatment had progressed to hypothyroidism, of which 7 (53.85%) presented aggravated eye symptoms. Twelve of 43 patients were smokers (all males), of which 10 (83.33%) had smoked for more than 10 years. The IOP was significantly higher in the DON patients compared to the other groups (*p* < 0.001) (Fig. 1). The data are summarized in Table 1. MRI pictures of four groups are presented in Fig. 2.

**Table 1:** Baseline data of all four groups

		DON group	NDON group	ANDON group	NC group	*P* value
*n*		6	37	39	52	–
Male ratio (%)		100	37.84	33.33	38.46	0.019^b^
Age (year)		46.67 ± 10.60	38.92 ± 11.31	47.13 ± 9.53	39.88 ± 12.17	0.007^a^
IOP (mmHg)		30.98 ± 8.18	17.48 ± 3.67	20.16 ± 3.00	15.44 ± 2.62	< 0.001^a^
Duration (month)		21.00 (7.00–24.00)	9.00 (2.00–36.00)	6.00 (3.00–12.00)	N/A	0.08^c^
Thyroid function (%)	hyperthyroidism	6(100%)	20(54.05%)	26(66.67%)	N/A	0.085^b^
	hypothyroidism	(0.00%)	10(27.03%)	8(20.51%)	N/A	0.385^b^
	normal thyroid function	(0.00%)	7(18.92%)	5(12.82%)	N/A	0.554^b^
Number of affected muscles		3.5 (3–4)	1 (0–1.5)	2 (1–4)	N/A	< 0.001^c^

N/A: not applicable for this group

^a^Welch’s ANOVA

^b^Fisher exact test

^c^Kruskal–Wallis test

^d^ANOVA; *P* < 0.05, there is significant difference among groups

### Peripapillary VD and RNFLT

**Fig. 3:**
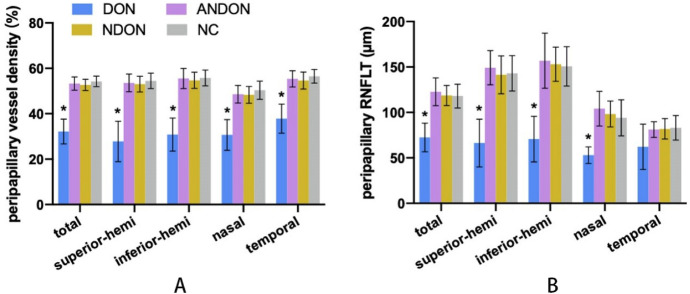
Evaluation of peripapillary VD **a** and RNFLT **b** in the four groups. (* *P* < 0.05 between indicated groups)

**Fig. 4:**
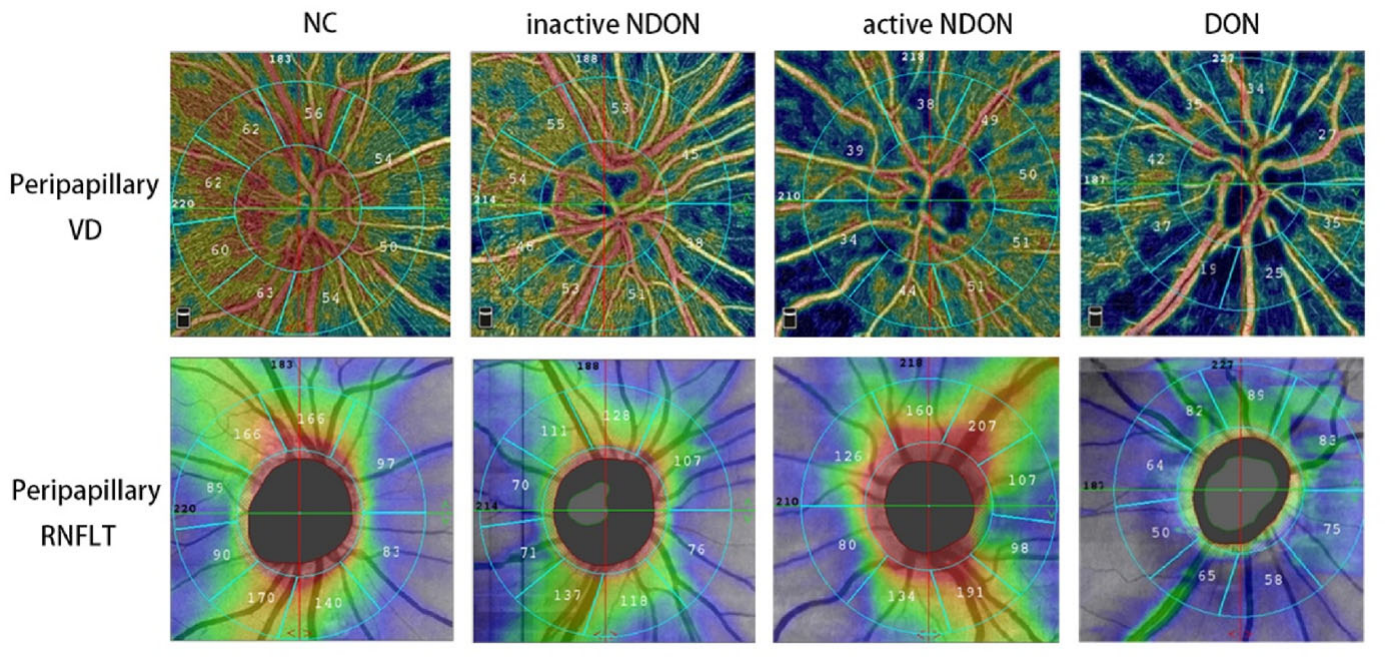
OCTA images of the four groups. Peripapillary VD and RNFLT of NC group were normal. NDON group showed minor decrease of peripapillary VD and RNFLT. ANDON and DON groups showed obviously diminished peripapillary VD

The OCTA images of all four groups were analyzed to determine peripapillary VD and RNFLT (Fig. 3, Tables 2 and 3). Total peripapillary VD of the DON group was only 32.18 ± 5.48% compared to 52.69 ± 2.48%, 53.31 ± 2.93% and 54.26 ± 2.30% recorded for the NDON, ANDON and NC groups, respectively (*P* < 0.001 for each pair comparison). Total peripapillary RNFLT in the DON group was 63.47 ± 15.81 μm, and was significantly lower compared to that in other groups (118.68 ± 11.08 μm, 122.79 ± 15.33 μm and 118.01 ± 13.07 μm for NDON, ANDON and NC groups respectively; *P* < 0.001 for each). Likewise, the superior, inferior and nasal sector peripapillary RNFLT in the DON patients showed significant differences with the corresponding values in the other groups (*p* < 0.001). However, total and regional RNFLT were similar among the NDON, ANDON, and NC groups. The OCTA images of different groups are shown in Fig. 4.

**Table 2:** Peripapillary VD of different groups

	DON group	NDON group	ANDON group	NC group	*P* value
Total peripapillary VD (%)	32.18 ± 5.48	52.69 ± 2.48	53.31 ± 2.93	54.26 ± 2.30	< 0.001^b^
Superior VD (%)	27.80 ± 8.93	53.03 ± 3.49	53.68 ± 3.89	54.50 ± 3.37	< 0.001^b^
Inferior VD (%)	30.83 ± 7.25	54.73 ± 3.56	55.54 ± 4.43	55.77 ± 3.48	< 0.001^b^
Nasal VD (%)	30.67 ± 6.74	48.35 ± 3.69	48.64 ± 3.85	50.35 ± 4.00	< 0.001^a^
Temporal VD (%)	37.83 ± 6.37	54.65 ± 3.72	55.36 ± 3.57	56.49 ± 2.98	< 0.001^b^

^a^ANOVA

^b^Welch’s ANOVA; *P* < 0.05, there is significant difference among the groups

**Table 3:** Peripapillary RNFLT of different groups

	DON group	NDON group	ANDON group	NC group	*P* value
Total peripapillary RNFLT (μm)	63.47 ± 15.81	118.68 ± 11.08	122.79 ± 15.33	118.01 ± 13.07	< 0.001^a^
Superior RNFLT (μm)	66.40 ± 26.29	141.41 ± 20.90	149.29 ± 18.86	143.06 ± 19.51	< 0.001^a^
Inferior RNFLT (μm)	70.67 ± 25.05	153.08 ± 18.88	156.90 ± 30.36	150.73 ± 21.65	< 0.001^a^
Nasal RNFLT (μm)	53.00 ± 9.06	98.30 ± 14.25	104.18 ± 19.04	94.10 ± 19.89	< 0.001^a^
Temporal RNFLT (μm)	62.17 ± 24.86	81.95 ± 11.27	81.18 ± 8.62	83.10 ± 13.54	0.274^b^

^a^ANOVA

^b^Welch’s ANOVA; *P* < 0.05, there is significant difference among the groups

### Factors associated with peripapillary VD and RNFLT

Single factor correlation analysis was conducted with peripapillary VD or RNFLT as the dependent variable, and gender, age, duration of TAO, smoking history, thyroid condition, proptosis degree, IOP, number of affected muscles as the independent variables. IOP, gender, thyroid condition and smoking history were significantly correlated to peripapillary VD and RNFLT, whereas proptosis degree was associated with peripapillary RNFLT (*P* < 0.05) (Table 4). Multivariable linear regression model further identified IOP as the only independent factor of peripapillary VD and RNFLT (*β* = −0.465, *P* < 0.001, adjusted *R*^2^ = 0.381 and *β* = −0.343, *P* = 0.002, adjusted *R*^2^ = 0.234 respectively; Table 5).

**Table 4:** Single factor correlation analysis of peripapillary VD and RNFLT

	VD		RNFLT	
	Correlation Coefficient	*P* Value	Correlation coefficient	*P* Value
IOP (mmHg)	− 0.277	0.012^a^	− 0.244	0.027^a^
Gender	− 0.395	< 0.001^b^	− 0.364	0.001^b^
Smoking history	− 0.463	< 0.001^b^	− 0.362	0.001^b^
Thyroid condition	− 0.231	0.037^b^	− 0.231	0.037^b^
Proptosis degree (mm)	− 0.065	0.559^a^	− 0.297	0.007^a^

^a^Spearman test for correlation; ^b^point-biserial correlation

**Table 5:** Multivariable linear regression analysis of peripapillary VD and RNFLT

	VD				RNFLT			
	*β*	*t*	*P*	Adjusted R Square	*β*	*t*	*P*	Adjusted R Square
IOP (mmHg)	− 0.465	− 4.822	< 0.001	0.381	− 0.343	− 3.198	0.002	0.234
Gender	− 0.194	− 1.328	0.188		− 0.269	− 1.555	0.124	
Smoking history	− 0.12	− 0.768	0.445		0.054	0.309	0.758	
Thyroid condition	− 0.063	− 0.667	0.507		− 0.12	− 1.115	0.269	
Proptosis degree (mm)	–	–	–		− 0.108	− 0.946	0.347	

## Discussion

DON is often characterized by irreversible orbital changes, such as extraocular muscles enlargement, fibrosis and fatty degeneration, which significantly increase the orbital volume and restrict the exophthalmos process. This eventually increases the IOP, resulting in compression of the orbital blood vessel and optic nerve [17–19]. Consistent with previous reports, the IOP of DON patients were significantly higher and peripapillary VD was significantly lower compared to that of the other groups. Multivariable linear regression model further confirmed that increased IOP is an independent factor of low peripapillary VD. Another study also reported a similar relationship between IOP and optic nerve head perfusion [18]. While pressure from enlarged extraocular muscles and orbital fat compress the eyeball, increased episcleral vein pressure caused by high orbital tension can lead to higher outflow resistance of aqueous humor, resulting in high IOP [19]. Therefore, the decrease in peripapillary VD might be the result of orbital blood vessel compression and increased IOP. The total peripapillary RNFLT was also markedly lower in the DON patients, and affected by the IOP. It is possible that hypoxic-ischemia resulting from increased IOP and the concomitant decrease in blood supply leads to the loss of retinal nerve cells. In addition, the RNFLT in the superior, inferior and nasal peripapillary areas were lower in the DON group, which might due to increased IOP in these susceptible areas.

Smoking was significantly correlated with peripapillary VD and RNFLT in our cohort, and TAO patients with a history of smoking had thinner RNFL and fewer retinal blood vessels compared to the non-smokers [20]. Previous studies have also established smoking as a risk factor for TAO progression [6, 21]. There is also evidence that peripapillary VD in active TAO is lower than that during inactive disease [15], although Lei Ye et all [14] reported increased macular VD in TAO patients. Furthermore, Kyle T Lewis et all [10] found that peripapillary VD decreased in DON patients after decompression surgery and improved the symptoms, whereas one patient showed deterioration of symptoms with increased peripapillary VD. However, we did not detect any significant difference between the peripapillary VD of active and inactive TAO patients, which could be attributed to the small sample size. Since peripapillary VD in the TAO eyes is easily affected by orbital and intraocular pressure and appears earlier than visual impairment [22], it is a reliable predictive indicator of DON and visual prognosis. However, differences in sample size, racial diversity, parameter settings of OCTA and inclusion criteria for TAO patients may have resulted in the discrepancies among previous studies. Future investigation is needed to validate the predictive and diagnostic potential of OCTA in TAO and DON.

There are several limitations in our study that ought to be considered. First, selection bias could not be ruled out since all recruited patients visited the hospital for ocular symptoms. In addition, we did not have the long-term follow-up data to evaluate the true predictive value of reduced peripapillary VD in TAO. Second, the sample size was relatively small, which limits the generalizability of the results. Furthermore, ocular blood supply is affected by multiple factors such as hypertension, and variations in VD across the different retinal areas may also have an effect on OCTA results. Therefore, these factors should be taken into consideration when analyzing the activity and severity of TAO by OCTA.

In conclusion, reduced peripapillary VD as measured by OCTA is a promising prognostic indicator of optic neuropathy in TAO and can diagnose early subclinical DON. High IOP should be considered a risk factor for decreased retinal vessel and nerve fiber layer thickness in TAO patients. Further studies should be conducted on larger cohorts to validate the present findings.

## Data Availability

The datasets used or analyzed during the current study are available from the corresponding author on reasonable request.
